# Restrictive but not restricted: Perspectives on antimicrobial use and antimicrobial resistance among Swedish dairy veterinarians

**DOI:** 10.1002/vro2.25

**Published:** 2021-12-27

**Authors:** Hedvig Gröndal, Nils Fall, Isabel Blanco‐Penedo, Susanna Sternberg‐Lewerin

**Affiliations:** ^1^ Department of Biomedical Sciences and Veterinary Public Health Swedish University of Agricultural Sciences Uppsala Sweden; ^2^ Department of Clinical Sciences Unit of Veterinary Epidemiology Swedish University of Agricultural Sciences Uppsala Sweden; ^3^ Department of Animal Science University of Lleida Lleida Spain

## Abstract

**Background and aims:**

In Europe, the antimicrobial use (AMU) for food‐producing animals has decreased rapidly. However, studies indicate that a too strict policy, with too restrictive AMU, is potentially problematic for veterinarians because it threatens animal welfare and creates tensions between farmers and veterinarians. The AMU in Sweden is among the lowest in Europe, and regulation of AMU in farm animals is strict. The aim of our study was to explore how Swedish veterinarians describe the relations between (1) being restrictive with antibiotics due to the risk of AMR and (2) concerns for animal welfare and/or the veterinarian‐client relationship.

**Methods:**

Semi‐structured interviews with 21 veterinarians, working with dairy cattle, were performed. The transcripts were analysed, and a number of dominant patterns which recurred in all, or most of, the interviews were identified.

**Result:**

The interviewed veterinarians described AMR prevention and tackling the threat AMR poses towards public health, as central for their profession and as influencing their everyday practice and decisions on AMU. Importantly, veterinarians described accounting for AMR in everyday practice as fairly unproblematic, both in relation to animal welfare as well as in relation to farmers. The veterinarians generally perceived that they could treat animals with antibiotics when justified, and being restrictive with antibiotics was described as an expression of professional skill and not as challenging as animal welfare. Moreover, they stated that restrictive AMU seldom or never caused conflicts with farmers.

**Conclusion:**

Strict AMU policy and restrictive AMU do not necessarily put veterinarians in a problematic position where they are caught between conflicting demands and risks.

## INTRODUCTION

Antimicrobial resistance (AMR) has made antimicrobial use (AMU) in farm animals a public concern and a matter in need of governance.^1^, ^2^ In Europe, following intense policy work, the AMU for food‐producing animals has decreased rapidly.^3^ However, studies indicate that veterinarians perceive that a too strict AMU policy, with too restrictive AMU, is potentially problematic because it threatens animal welfare and creates tensions between farmers and veterinarians.^4^, ^5^, ^6^, ^7^, ^8^, ^9^ Studies report that AMU policies risk putting veterinarians in a difficult position where, on the one hand, they should account for the future risk AMR poses for public health and, on the other hand, have other more urgent responsibilities towards animal welfare and farmer demands.^4^, ^5^, ^6^, ^7^, ^8^, ^9^ How do veterinarians in countries with strict policies and low AMU experience their position in relation to AMU? Do these veterinarians perceive tensions between following AMU policies and treating animals in need of antibiotics? Do they feel restricted by the policy in their everyday work? Moreover, does adherence to policy lead to conflicts between veterinarians and farmers?

The AMU in Sweden is among the lowest in Europe. In addition, the most used antibiotic substance is penicillin^10^ which as a narrow‐spectrum antibiotic is less prone to cause AMR than broad‐spectrum antibiotics. The low AMU in Sweden is the result of intense policy work since the 1980s. The Swedish regulation of AMU in farm animals is strict from a European perspective, and AMR is a prioritised matter in Swedish governmental policy.^10^, ^11^

Previous studies report that veterinarians in general are well aware of AMR and that they see themselves as having responsibility for protecting public health against AMR.^4^, ^5^, ^8^, ^9^, ^12^, ^13^, ^14^ However, several of the same studies also report that there is scepticism among some veterinarians towards the connection between AMU for animals and AMR‐related risks for humans. Such scepticism might decrease veterinarians’ motivation to be restrictive with antibiotics.^4^, ^14^ Even if livestock veterinarians in general are aware of AMR and want to be restrictive with antibiotics, various studies show that implementing this awareness in everyday practice is not easy. Veterinarians have to consider not only the risk AMR poses for the general public, they also have to consider other risks and interests; sometimes these can be in conflict with each other (cf. 8,9). One report^7^ described veterinarians as being placed in a 'double bind' due to the relations between antibiotics, animal well‐being and human health, which do not always align well. Another study^5^ argued that pig veterinarians in the UK experienced an ethical conflict between their social responsibility for reducing AMU and ensuring pig health and welfare.^4^, ^6^, ^9^

Importantly, studies indicate that when veterinarians need to balance the more abstract AMR risk against current and more salient risks and interests, the latter tend to be prioritised. In a previous study,^9^ the informants expressed that due to their veterinary duty towards animal welfare, they need to treat animals in need of antibiotics, regardless of the risk for AMR. Another study^13^ argued that even though veterinarians and farmers understand and acknowledge AMR‐related risks on a conceptual level, there is a conflict between this and the actual AMU behaviour. Thus, in actual practice, current concerns (such as animal welfare, productivity and the relationship to the farmer) tend to be prioritised by the veterinarians over the (theoretical and future) risk for AMR. Studies report that this state of affairs is amplified by the fact that AMR is seldom experienced as a problem in veterinarians’ clinical practice; even when treatment failures do occur, veterinarians seldom attribute them to resistance.^5^, ^6^, ^9^

Previous studies raised questions concerning how Swedish veterinarians perceive the comparably strict AMU regulation and low AMU – do they feel that the restrictive use is in conflict with their obligation to secure animal welfare, and how does the strict AMU policy influence their relation to farmers? Interestingly, an interview study of Swedish dairy farmers^15^ reported that these did not find the comparably strict regulations on AMU as causing a lack of access to needed antibiotics, or a threat to animal welfare. Instead, these farmers seemed to find the Swedish AMU policy appropriate.

The aim of this study was to explore Swedish veterinarians’ perspectives on AMU for dairy cattle in relation to different risks and/or demands. Specifically, we aimed to explore how veterinarians describe the relations between (1) being restrictive with antibiotics due to the risk of AMR and (2) concerns for animal welfare and/or the veterinarian‐client relationship.

## METHODS

### Participants

Recruitment of veterinarians was performed through calling veterinary practices and sending further information to veterinarians who had shown an interest in participating. Participants were informed that participation in the study was voluntary and that data would be anonymized. Eventually, 21 veterinarians were recruited (see Table 1). The recruited veterinarians worked in different locations in the middle and south of Sweden. Both publicly employed and private/self‐employed livestock veterinarians and both veterinarians working with mixed species and only livestock were recruited. The vast majority of the recruited veterinarians were women, this reflects the fact that 80% of Swedish livestock veterinarians are women. While around 60% of the new Swedish veterinarians are educated abroad, all of the interviewed veterinarians were educated in Sweden.^16^

**TABLE 1:** Overview of participants in the study

	Practice type	Worked for	Gender	Main animal species treated
1.	Private	+30 years	Woman	Mixed
2.	Public	+2 years	Woman	Mixed, mostly cattle
3.	Public	+20 years	Woman	Mixed, mostly large animals
4.	Public	+2 years	Woman	Mixed, mostly large animals
5.	Private	+5 years	Man	Mixed, majority large animals
6.	Public	+10 years	Woman	Mixed, mostly horses.
7.	Public	+10 years	Woman	Only cattle
8.	Private	+20 years	Woman	Mixed, mostly cattle
9.	Private	+25 years	Woman	Mixed, mostly cattle
10.	Public	+5 years	Woman	Mixed
11.	Private	10 years	Woman	Only large animals
12.	Private	+10 years	Woman	Only large animals
13.	Public	+5 years	Man	Mixed
14.	Public	+30 years	Woman	Mixed
15.	Private	10 years	Woman	Only large animals
16.	Public	2 years	Woman	Mixed, mostly small animals
17.	Public	10 years	Woman	Mixed, mostly cattle
18.	Public	+20 years	Woman	Mixed
19.	Public	>1 year	Woman	Mixed, mostly large animals
20.	Private	+20 years	Man	Mixed
21.	Private and public	+5 years	Woman	Only dairy cattle

The interviews were performed by the first author during 2020. The interviews were planned to be face to face; however, they were conducted remotely over the telephone (except the first one) due to the COVID‐19 pandemic. The interviews took between 45 min and 1.5 h. Interviews were semi‐structured and contained both questions on diagnostic practice, decisions on AMU and more general questions about AMU and AMR. The semi‐structured interview meant that an interview guide was used but that the exact content of each interview differed slightly. The interviews were recorded and transcribed by the first author. Data were stored in a storage platform. The interviews were performed in Swedish; the authors translated the quotes cited in the result section after the analysis.

### Analytical process

The analysis was performed manually. First, all interviews were read, and an initial descriptive coding^17^, ^18^ was performed. This coding was empirically driven; however it started from a general research interest in how AMU, AMR and AMU policies were described by the veterinarians. In the first step, a range of codes were created. The second step focused on codes that referred to how the interviewees described AMU in relation to AMR, animal welfare and farmer relations. In this step, interviews were re‐read and codes adjusted. Here, a number of more abstract patterns or themes^17^ recurring in many or all interviews emerged. Statements explicitly opposing the themes exist, although they are rare in the data. The recurring themes were the focus of the current study and are presented below in the results section with some illustrative quotes (an extended set of quotes is given in Supporting Information S1).

## RESULTS

### AMR and restrictive AMU as a prioritised matter influencing everyday practice

Throughout the interviews, AMR and prevention of AMR were described as something the veterinarians were well aware of and as an important concern for them as professionals. Most veterinarians described AMR and careful AMU as something that they perceive as prioritised among Swedish veterinarians in general. The more junior veterinarians stated that AMR and the connection between AMU and AMR had been a central topic in their education.

**Veterinarian**: Well, if you consider the actual development of resistance, I definitely believe that, I could phrase it like this, that it is an attitude, I guess, within the Swedish veterinary profession, because we are really good at being careful with antibiotics. (Interview 6, public practice, worked for +10 years).

**Interviewer**: But how is it that, do you think, that you as a veterinarian, that you feel that responsibility, like, that you feel that, well, that you feel that you have to account for that?

**Veterinarian**: Well, they repeated it throughout the education, that probably matters. (…) But also that, I feel that I cannot enter any veterinarian forum without AMR being addressed in one way or another. (Interview 2, public practice, worked +2 years).

A recurring theme in the interviews was that veterinarians described that their concern for AMR influences their everyday professional practice. Thus, their concerns for AMR were not only conceptual but had consequences for their decisions on antibiotics.

**Interviewer**: Is it, would you say that (antimicrobial resistance) is present as a factor, well, when you presc…when you consider antibiotics? That you sort of… if the problems with resistance did not exist… do you believe that you would prescribe more?

**Veterinarian**: Yes, indeed! Then it would have been really easy to treat all cows with sub‐clinical mastitis with penicillin… Everybody with a fever would get antibiotics. (…) I would mess around with Baytril (a quinolone). Everyone, everyone lying down and that has a bad udder gets Baytril if I do not have to care about resistance. (Interview 2, public practice, worked +2 years).

When asked about their main concerns related to AMR, most veterinarians described the threat AMR poses to human health as most central for them. While some veterinarians referred to a combination of human and animal health as their main concern, none of the interviewed veterinarians described the risk AMR poses for animal health as their only concern. Thus, throughout the interviews, the veterinarians described themselves as having a professional responsibility for the health of the future human population.

**Interviewer**: One last question, if you think like this, what do you believe is most central for a responsible use of antibiotics for the cows? What is most important for you?

**Veterinarian**: Yes, but that is actually our… It is human health, it really is, and then also we do not want increasing resistance. It will affect the cows as well if there is resistance, at first, but in the next step it can affect humans as well.

The veterinarians were in general well aware of existing national AMU guidelines. These guidelines were described as credible and useful.

**Veterinarian**: The district veterinarians (government organisation) in general have our treatment guidelines and such that we take into account.

**Interviewer**: Do you use them in your daily work?

**Veterinarian**: Yes I do. They are based on science and proven experience; it is our skilled head veterinarians that compile them so I use them a lot. (Interview 19, public practice, worked less than 1 year)

The more experienced veterinarians (>10 years), however, commonly said that the guidelines were not something that they needed to read on a daily basis, but they described that their AMU in general was in accordance with the guidelines and that they sometimes read the guidelines to become updated.

#### Restrictive AMU as an effective treatment and a sign of skill

A recurring theme in our interviews was that the veterinarians described a restrictive use of antibiotics and adherence to AMU policies, as aligning well with good veterinary care and animal welfare. As an example, several of the interviewed veterinarians described how the use of quinolones had been restricted by the authorities, in order to save the quinolones for human use. This policy change was referred to as something that the veterinarians supported. Central for this support was however that the veterinarians were convinced that quinolones had no significant effect on *E. coli* mastitis and that anti‐inflammatory drugs and frequent milking were more effective.

**Veterinarian**: And, also, enrofloxacin or Baytril, we did use that for coli mastitis, but that is also broad spectrum; it should not be used. And new results have shown that it is not effective for coli mastitis. (Interview 9, public practice, worked +25 years).

Importantly, the veterinarians described that they could effectively treat animals in need of antibiotics:

**Interviewer**: Well, consider this, generally speaking: Do you feel, in your practice, do you ever feel constrained by the somewhat restrictive antibiotic policy in Sweden? Do you feel constrained by this?

**Veterinarian**: No, I actually do not think so. No.

**Interviewer**: You never feel like… well, feel like, this animal… that it can be at the expense of good veterinary care?

**Veterinarian**: No, actually, no I do not think so. I think, not regarding antibiotics. In relation to other aspects, I might lack treatment alternatives and so on, but in regard to antibiotics I feel that, I think that is not a problem. It is very seldom that I feel… It has not even crossed my mind that 'oh God, what if I had that kind of antibiotics, in that case…' No I think they… No I do not think that.

**Interviewer**: And you feel that you can treat the ones that need it?

**Veterinarian**: Yes, I think so, I think so. With good results. (Interview 11, private practice, worked +10 years).

Previously in this interview, this veterinarian described that she almost exclusively used penicillin when treating cows. However, she did not experience that this prescribing practice was at the expense of good veterinary care or effective treatment. Important to note, however, is that Sweden has a favourable AMR situation with most Gram‐positive mastitis pathogens still susceptible to penicillin, which is the first‐hand choice of treatment when such bacteria are suspected in mastitis cases.^19^

Being careful with antibiotics was by several veterinarians framed in terms of professional pride and as opposed to taking (unprofessional) short‐cuts. They describe that use of narrow‐spectrum antibiotics, and finding other treatment alternatives than antibiotics, often require precise diagnostics and veterinary expertise.

**Veterinarian**: Yes, for me it is also a matter of prestige, to treat what you actually know with…rather, how you could frame it, precision. To close your eyes and aim widely/broadly, then, anyone can do that. (Interview 14, public practice, worked +5 years).

In several interviews, being restrictive with antibiotics – and accounting for AMR, was thus framed as compatible with effective treatment in terms of certain forms of prescribing (e.g., precise and primarily prescribing drugs with a narrow‐spectrum) and other kinds of treatment of animals with signs. A non‐restrictive prescribing was instead framed as an expression of lack of skills and taking professional shortcuts, rather than a way to protect animal welfare.

#### Restrictive AMU and good relationships with farmers

Restrictive use of antibiotics was generally not described as problematic in relation to farmers. Throughout the interviews, the veterinarians described conflicts with farmers related to AMU and farmers that explicitly demand antibiotics, or certain kinds of antibiotics, as uncommon.

**Interviewer**: How…you said that in general, you, you and the animal owners agree, is that also true in regard to antibiotic prescribing? Do you usually agree when it is needed and not needed?

**Veterinarian**: Yes.

**Interviewer**: Has it happened, that you disagree?

**Veterinarian**: Actually, I cannot think of any situation when it has happened, so I guess I can say no (Interview 1, private practice, worked +30 years).

**Interviewer**: And how do you, how do you think, if you say that 'no, we should wait, I want to perform a culture' or…How do you think they (the farmers) would react?

**Veterinarian**: I think they are quite well‐behaved around here. I entered a pretty good group of younger colleagues who has… paved the way for me to get…

**Interviewer**: So, it is okay to say no? (to antibiotics)

**Veterinarian**: Yes, I think so, I have never experienced that someone has, you know, become angry, or tried to convince me. No, no. (Interview 11, private practice, worked for 10 years).

**Veterinarian**: but I still think that, I think that the farmers are like the public, everyone is aware of resistance and that, when you talk about, so most buy it and understand it. Like, it is the stuff we want to save for human healthcare. (Interview 3, public practice, worked for 20 years).

As in these examples, the veterinarians generally described farmers as well‐educated concerning when antibiotics are needed. Thus, they stated that they are mostly called out to farms when antibiotic treatments are actually justified, and this decreases the risks of conflicts. Several of the more experienced veterinarians described that this state of affairs is a consequence of having worked with the same farmers for many years. The long‐standing relationships have not only led to farmers knowing when antibiotics might be needed but also to a more general trust in the veterinarian. In addition, the veterinarians described farmers as well aware of AMR and concerned by the risk AMR poses for human health. Thus, veterinarians described that they are able to practice and maintain restrictive antibiotic use with little resistance from clients.

## DISCUSSION

### Restrictive AMU as fairly unproblematic

The aim of this study was to explore Swedish veterinarians’ perspectives on AMU in dairy cattle, in relation to different kinds of risks and/or demands. The analysis shows that interviewed veterinarians described AMR prevention and tackling the threat AMR poses to public health through restrictive AMU, as central for their profession. Similar findings have been made in other studies outside Sweden.^4^, ^8^, ^9^, ^12^, ^14^ However, while previous studies report that in practice the concern for AMR tends to be out‐ruled by more urgent matters as animal welfare and farmer demands,^5^, ^13^ the veterinarians in our study described their concerns about AMR as influencing actual practice and decisions on antibiotics.^4^, ^5^, ^6^, ^8^, ^9^ While previous studies indicated that this would put the veterinarians in a problematic position, our informants did not perceive themselves as placed in a double bind^7^ or ethical conflict^5^ due to conflicting demands. Instead, being restrictive in AMU was framed as a fairly unproblematic matter; something that was more or less taken for granted in the profession and something that did not evoke a lot of tensions. Since European AMU for livestock animals has decreased rapidly in years, it is important to explore if this finding is exclusive to the Swedish (and Nordic) context or if it mirrors a broader development, which was not yet evident when earlier studies were published.

Most importantly, our study showed that animal welfare, in terms of efficient treatment generally aligned well with being restrictive with antibiotics according to the veterinarians. This finding should be seen in the context of an already existing focus on preventive animal healthcare leading to low disease prevalence, low AMU and good welfare. The restrictive AMU policy is hence not perceived as overly restrictive but rather as preserving the effectiveness of antibiotics. A dominant pattern in the interviews was that the veterinarians felt they could combine their professional duties regarding precise diagnosis and efficient treatment, with their duties towards future human health. Thus, while previous studies reported that accounting for AMR and accounting for animal welfare in practice become opposites, our study showed that it can be different and that AMR prevention and animal welfare can actually be aligned.

Our results complement a previous Swedish study^15^ where dairy cattle farmers agreed on the strict Swedish AMU policy, that farmers trusted their veterinarian and that farmers felt that their animals received sufficient antibiotic treatment. The current and the previous study^15^ indicate that AMU is not a common matter for conflicts between veterinarians and dairy cattle farmers in Sweden. Furthermore, it was reported that Swedish farmers’ perspectives on and practices of AMU appear to have 'over time been shaped by the Swedish strict regulation.'^15^ Our results indicate that this is also the case with Swedish veterinarians.

The finding that the veterinarians saw professional AMU guidelines as credible and usable, that they described themselves as loyal to the general Swedish policy on AMU, partly differs from findings in other countries. Studies indicate that AMU guidelines tend not to be used by veterinarians or at least that guidelines are not a key source of information for veterinarians when prescribing.^5^, ^12^ Some studies have indicated that Swedish veterinarians may differ from veterinarians in other countries in that they refer to AMU guidelines as the most important source of information when prescribing.^12^ Concerning government policy goals for AMU, one study^9^ reported that Dutch veterinarians were sceptical about the national policy goal to reduce livestock AMU by 50%. The veterinarians feared that the goal would mean animals in need of antibiotics would not be treated, and they questioned the science behind the policy.^8^ It appears that veterinarians in this national context, at this time, were more sceptical about the national AMU policy than the veterinarians in our study who described themselves as loyal to the more restrictive Swedish policy. That study^9^ is 6 years old, and it is possible that the results would be different now. It is also important to note that since veterinary AMU is already low in Sweden, and AMU regulations are strict, there are currently no ambitions to drastically reduce the AMU further. Thus, the veterinarians in our study could probably reflect on their AMU without feeling that this is particularly questioned nor threatened. As discussed above, a key for the veterinarians' approach to AMU appeared to be that they felt they could treat their patients in an efficient manner. It should be noted that such approaches would not include alternative medicines (i.e., treatments outside classical medicine, e.g., homeopathy) because Swedish legislation does not allow veterinarians to use such products. As a consequence, their relation to the restrictive AMU policy appears to be rather uncomplicated and not ambiguous as previously reported.^13^ However, it is highly probable that they would be sceptical about a policy that radically limited their current AMU, which they found justifiable. Moreover, it is important to note that AMR‐rates in Sweden are low and that penicillin is effective for treating most infections in cattle.^20^ In addition, since the 1980s, there has been a strong focus on prevention of infections among livestock.^21^

In this study, we have reported on the most dominant patterns identified in the interviews. There are statements that diverge from these patterns. These will be analysed further and addressed in future work. The main limitations of the current study can be related to the sample of veterinarians and the methodology. All the interviewed veterinarians were educated in Sweden, and it is possible that veterinarians educated in other countries may have other perspectives on AMU and AMR. In addition, it is possible that the veterinarians, when they self‐report on their practice, exaggerate their concern for AMR and how it influences practice. Moreover, it might be that the veterinarians who chose to take part in the study are more engaged in AMR and AMU than the average Swedish veterinarian. This limitation, however, also applies to previous studies on the subject. We argue that the qualitative interview study has important strengths. It can produce complex and detailed knowledge which cannot be retrieved thorough for example a survey study. In order to gain more knowledge on veterinarians’ AMU practices both qualitative studies, drawing on ethnography and participant observations, and quantitative audit studies of prescribing practices would be beneficial.

Our results indicate that several separate and partly interrelated factors mean that restrictive AMU for dairy cattle is a fairly unproblematic matter of course in Sweden: (1) The notion that AMR prevention and AMR‐related human health risks are within the veterinarians’ professional responsibility. (2) A feeling that this is currently agreed on within the profession (in Sweden). (3) A notion that the current AMU is restrictive. (4) Experience of AMU policies as credible and useful. (5) Professional space to effectively treat animals (also animals in need of antibiotics). (6) A notion of precise AMU as an expression of advanced veterinary skill and (7) Good relations with farmers despite restrictive use. It is probably through the combination of these factors that accounting for the AMR‐related public health risk (in an abstract future) and restrictive use of antibiotics can have such a strong position. Thus, just as non‐restrictive or 'misuse' of antibiotics cannot be reduced to a matter of individual behaviour, but is something that needs to be understood in its specific context^4^, ^15^; our study also demonstrates this to be central for restrictive AMU by Swedish livestock veterinarians.

## CONFLICT OF INTEREST

All authors declare they have no conflict of interest.

## ETHICS STATEMENT

According to the Swedish Ethical Authority, the study did not require ethical approval because no sensitive personal information was collected (reference ‐ law on ethical review of research involving humans 2003; available at https://www.riksdagen.se/sv/dokument-lagar/dokument/svensk-forfattningssamling/lag-2003460-om-etikprovning-av-forskning-som_sfs-2003-460; accessed 08/11/2021).

## Supporting information

Supporting information

Supporting information

## Data Availability

The authors elect to not share data.
