# Supplementary material for: Restrictive but not restricted: Perspectives on antimicrobial use and antimicrobial resistance among Swedish dairy veterinarians
Source: Vet Rec Open. 2021 Dec 27;8(1):e25. doi: 10.1002/vro2.25 (PMC8711788; doi:10.1002/vro2.25)
Supplement: Supplementary file 1 — Supporting information [file VRO2-8-e25-s001.docx]

# **Supporting Information S1**

Extended results section with more responses from participants.

## Results

### AMR and restrictive AMU as a prioritised matter influencing everyday practice

Throughout the interviews, AMR and prevention of AMR, was described as something the veterinarians were well aware of and as an important concern for them as professionals. Most veterinarians described AMR and careful AMU as something that they perceive as prioritised among Swedish veterinarians in general. The more junior veterinarians stated that AMR and the connection between AMU and AMR had been a central topic in their education.

**Veterinarian:** Well, if you consider the actual development of resistance, I definitely believe that, I could phrase it like this, that it is an attitude, I guess, within the Swedish veterinary profession, because we are really good at being careful with antibiotics. (Interview 6, public practice, worked for + 10 years).

**Interviewer:** But how is it that, do you think, that you as a veterinarian, that you feel that responsibility, like, that you feel that, well, that you feel that you have to account for that?

**Veterinarian:** Well, they repeated it throughout the education, that probably matters. […] But also that, I feel that I cannot enter any veterinarian forum without AMR being addressed in one way or another. (Interview 2, public practice, worked + 2 years).

A recurring theme in the interviews was that veterinarians described that their concern for AMR influences their everyday professional practice. Thus, their concerns for AMR were not only conceptual but had consequences for their decisions on antibiotics. Two examples from the interviews are given in S1.

**Interviewer:** Is it, would you say that [antibiotic resistance] is present as a factor, well, when you presc…when you consider antibiotics? That you sort of… if the problems with resistance did not exist… do you believe that you would prescribe more?

**Veterinarian:** Yes, indeed! Then it would have been really easy to treat all cows with sub-clinical mastitis with penicillin… Everybody with a fever would get antibiotics. […] I would mess around [ed] with Baytril [a quinolone]. Everyone, everyone lying down and that have a bad udder gets Baytril if I don’t have to care about resistance. (Interview 2, public practice, worked + 2 years).

**Interviewer:** Would you say that you, when you prescribe antibiotics, like, do you relate to the issue of AMR when you prescribe? Or is it something a bit distant?

**Veterinarian:** No, it is very close at hand, I think, when considering antibiotics. I really think I carry it with me in everyday practice… always when you consider antibiotics you have it in your mind, I have anyway. (Interview 17, public practice, worked for 10 years).

When asked about their main concerns about AMR, most veterinarians described the threat AMR poses to human health as most central for them. While some veterinarians referred to a combination of human and animal health as their main concern, none of the interviewed veterinarians described the risk AMR poses for animal health as their only concern. Thus, throughout the interviews, the veterinarians described themselves as having a professional responsibility for the health of the future human population.

**Interviewer:** One last question, if you think like this, what do you believe is most central for a responsible use of antibiotics for the cows? What is most important for you?

**Veterinarian:** Yes, but that is actually our… It is human health, it really is, and then also we do not want increasing resistance. It will affect the cows as well if there is resistance, at first, but in the next step it can affect humans as well.

**Interviewer:** And you think about that, you take that step into account?

**Veterinarian:** Absolutely, absolutely. If it was not because of human health, you would probably reason in a different way in relation to the cows, definitely. Because then it would have been comfortable to just provide antibiotics and not have to milk as much. […]. But for the sake of humans we cannot do that. So it is solely for the sake of humans, we could say, and a little for the cows because they would also be affected by resistance eventually, if it was used too generously. (Interview 18, public practice, worked for + 20 years).

**Interviewer:** But when you think about resistance as a factor that matters, do you think first of the risk for people, or the risk that you as a vet won’t be able to treat your…

**Veterinarian:** No, I probably think most about the human aspects, that we won’t be able to treat newborn children, you know, […] a tiny newborn baby, it’s for these children we do this, like, it’s a collective responsibility, that you can’t, you can’t justify that a cow would be more important. Actually. (Interview 7, public practice, worked for + 10 years)

The veterinarians were in general well aware of existing national AMU guidelines. These guidelines were in described as credible and useful.

**Veterinarian:** the district veterinarians [government organisation] in general have our treatment guidelines and such that we take into account.

**Interviewer:** do you use them in your daily work?

**Veterinarian:** Yes I do. They are based on science and proven experience, it’s our skilled head veterinarians that compile them so I use them a lot. (Interview 19, public practice, worked less than 1 year)

The more experienced veterinarians (>10 years) however, commonly said that the guidelines were not something that they needed to read on a daily basis, but they described that their AMU in general was in accordance with the guidelines and that they sometimes read the guidelines to become updated.

***Restrictive AMU as an effective treatment and a sign of skill***

A recurring theme in our interviews was that the veterinarians described a restrictive use of antibiotics, and adherence to AMU policies, as aligning well with good veterinary care and animal welfare. As an example, several of the interviewed veterinarians described how the use of quinolones had been restricted by the authorities, in order to save the quinolones for human use. This policy change was referred to as something that the veterinarians supported. Central for this support was however that the veterinarians were convinced that quinolones had no significant effect on *E. coli* mastitis , and that anti-inflammatory drugs and frequent milking were more effective.

**Veterinarian**: and, also, enrofloxacin or Baytril, we did use that for coli mastitis but that’s also broad spectrum, it shouldn’t be used. And new results have shown that it isn’t effective for coli mastitis. (Interview 9, public practice, worked + 25 years)

Importantly, the veterinarians’ described that they could effectively treat animals in need of antibiotics:

**Interviewer:** Well, consider this, generally speaking: Do you feel, in your practice, do you ever feel constrained by the somewhat restrictive antibiotic policy in Sweden? Do you feel constrained by this?

**Veterinarian:** No, I actually do not think so. No.

**Interviewer:** You never feel like… well, feel like, this animal… that it can be at the expense of good veterinary care?

**Veterinarian:** No, actually, no I do not think so. I think, not regarding antibiotics. In relation to other aspects, I might lack treatment alternatives and so on, but in regard to antibiotics I feel that, I think that’s not a problem. It is very seldom that I feel… It hasn’t even crossed my mind that “oh God, what if I had that kind of antibiotics, in that case…” No I think they… No I do not think that.

**Interviewer:** And you feel that you can treat the ones that need it?

**Veterinarian:** Yes, I think so, I think so. With good results. (Interview 11, private practice, worked + 10 years).

Previously in this interview, this veterinarian described that she almost exclusively used penicillin when treating cows. However, she did not experience that this prescribing practice was at the expense of good veterinary care nor effective treatment. Important to note, however, is that Sweden has a favourable AMR situation with most gram-positive mastitis pathogens still susceptible to penicillin, which is the first-hand choice of treatment when such bacteria are suspected in mastitis cases (19).

Another similar example follows:

**Interviewer**: Is there anything that could, that you experience as problematic with being restrictive, are there moments when it is at the expense of something?

**Veterinarian:** Of the animal welfare or so?

**Interviewer**: Yes, for example.

**Veterinarian:** No, I cannot say that. You know, I still have my own distinctions, I have my nowledge in veterinary medicine and I make my decisions based on that, so when I see that the animal should have antibiotics, I treat with antibiotics. So I cannot say that I think either the animal or the owner would suffer because of the decisions I make (Interview 6, public practice, worked for +10 years).

Being careful with antibiotics was by several veterinarians framed in terms of professional pride and as opposed to taking (unprofessional) short-cuts. They describe that use of narrow-spectrum antibiotics, and finding other treatment alternatives than antibiotics, often require precise diagnostics and veterinary expertise

**Veterinarian**: Yes, for me it is also a matter of prestige, to treat what you actually know with…rather, how could you frame it, precision. To close your eyes and aim widely/broadly, then, anyone can do that. (Interview 14, public practice, worked +5 years).

In several interviews, being restrictive with antibiotics – and accounting for AMR, was thus framed as compatible with effective treatment in terms of certain forms of prescribing (e.g. precise and primarily prescribing drugs with a narrow-spectrum), and other kinds of treatment of animals with symptoms. A non-restrictive prescribing was instead framed as an expression of lack of skills and taking professional shortcuts, rather than a way to protect animal welfare.

***Restrictive AMU and good relationships with farmers***

Restrictive use of antibiotics was generally not described as problematic in relation to farmers. Throughout the interviews, the veterinarians describe conflicts with farmers related to AMU and farmers that explicitly demand antibiotics, or certain kinds of antibiotics, as uncommon.

**Interviewer:** How…you said that in general, you, you and the animal owners agree, is that also true in regard to antibiotic prescribing? Do you usually agree when it is needed and not needed?

**Veterinarian:** Yes.

**Interviewer:** Has it happened, that you disagree?

**Veterinarian:** Actually, I cannot think of any situation when it has happened, so I guess I can say no (Interview 1, private practice, worked + 30 years)

**Interviewer**: And how do you, how do you think, if you say that “no, we should wait, I want to perform a culture” or…How do you think they [the farmers] would react?

**Veterinarian:** I think they are quite well-behaved around here. I entered a pretty good group of younger colleagues who has… paved the way for me to get…..[…]

**Interviewer:** So it is okay to say no? [to antibiotics]

**Veterinarian:** Yes, I think so, I have never experienced that someone has, you know, become angry, or tried to convince me. No, no. (Interview 11, private practice, worked for 10 years).

**Veterinarian:** but I still think that, I think that the farmers are like the public, everyone is aware of resistance and that, when you talk about, so most buy it and understand it. Like, it’s the stuff we want to save for human healthcare. (Interview 3, public practice, worked for 20 years)

As in these examples, the veterinarians generally described farmers as well-educated concerning when antibiotics are needed and not. Thus, they stated that they are mostly called out to farms when antibiotic treatments are actually justified and this decreases the risks of conflicts. Several of the more experienced veterinarians described that this state of affairs is a consequence of having worked with the same farmers for many years. The long-standing relationships have not only led to farmers knowing when antibiotics might be needed but also to a more general trust in the veterinarian. In addition, the veterinarians described farmers in terms as well aware of AMR, and concerned by the risk AMR poses for human health. Thus, veterinarians described that they are able to practice and maintain restrictive antibiotic use with little resistance from clients
